# Emotional Overeating during the COVID-19 Pandemic: Polish Adolescents’ COVID-19 Experience (PLACE-19) Study

**DOI:** 10.3390/nu15173818

**Published:** 2023-08-31

**Authors:** Dominika Głąbska, Dominika Skolmowska, Dominika Guzek

**Affiliations:** 1 Department of Dietetics, Institute of Human Nutrition Sciences, Warsaw University of Life Sciences (SGGW-WULS), 159C Nowoursynowska Street, 02-776 Warsaw, Poland; dominika_glabska@sggw.edu.pl (D.G.); dominika_skolmowska@sggw.edu.pl (D.S.); 2 Department of Food Market and Consumer Research, Institute of Human Nutrition Sciences, Warsaw University of Life Sciences (SGGW-WULS), 159C Nowoursynowska Street, 02-776 Warsaw, Poland

**Keywords:** emotional eating, emotional overeating, Emotional Overeating Questionnaire (EOQ), adolescents, body mass, body mass change, national study, COVID-19, PLACE-19 Study

## Abstract

Emotional overeating is the most frequently noted type of emotional eating, being commonly associated with increased consumption of energy-dense products, as well as excessive body mass, and weight gain. Even though a number of studies assessed emotional overeating during the COVID-19 pandemic in adult populations, studies of children and adolescents are scarce. The aim of the present study was to assess emotional overeating background, including consumption in response to six emotions (anxiety, sadness, loneliness, tiredness, anger, and happiness), in the population of Polish adolescents within the PLACE-19 Study during the COVID-19 pandemic. The PLACE-19 Study is a national Polish population-based study of adolescents gathered upon recruitment based on a random quota sampling of secondary schools, conducted in a population of 1126 students (818 females and 308 males, a median of age 17.0 and 16.5 years, respectively). Emotional overeating was assessed while using the Emotional Overeating Questionnaire (EOQ), and as additional factors, the following were assessed: gender, body mass, body mass change during the COVID-19 pandemic, and declared tempting food products. Female participants declared a higher frequency of overeating in response to feelings of anxiety, sadness, loneliness, and happiness, and were characterized by a higher total score than male participants, while *p* ≤ 0.05 was interpreted as a statistical significance. Obese participants declared a higher frequency of overeating in response to feelings of sadness, and loneliness than normal weight participants. Participants gaining weight declared a higher frequency of overeating in response to feelings of anxiety, sadness, loneliness, tiredness, and anger, and were characterized by a higher total score than participants losing weight or maintaining a stable weight, while participants gaining weight declared a higher frequency of overeating in response to feelings of happiness than participants losing weight. Participants declaring both sweet and salty products as tempting declared a higher frequency of overeating in response to feelings of anxiety, and sadness than participants declaring no tempting products; participants declaring both sweet and salty products declared a higher frequency of overeating in response to feelings of tiredness than participants declaring only salty products and those declaring no tempting products, as well as declared a higher frequency of overeating in response to feelings of happiness than participants declaring only sweet products, and those declaring no tempting products; participants declaring sweet products declared a higher frequency of overeating in response to feelings of anger than participants declaring no tempting products, while participants declaring both sweet and salty products declared a higher frequency of overeating in response to feelings of loneliness, and were characterized by a higher total score than all other respondents. The sub-groups with the highest frequency of emotional overeating were the female respondents, obese participants, those gaining weight, and those declaring both sweet and salty products as tempting, while among the emotions most often causing emotional overeating, there were sadness and loneliness.

## 1. Introduction

Emotional eating is in general defined as any food consumption in response to any emotion which is experienced, but traditionally only negative emotions as a stimulus, and overconsumption as an effect are taken into account [1]. However, it should be emphasized that the broad definition of emotional eating specifies that emotional eating may be classified as negative emotional eating (motivated by negative emotions, such as low mood, and associated with an attempt to manage experienced emotions), and positive emotional eating (motivated by positive emotions, such as happiness, and commonly associated with celebrations, and socializing) [2]. At the same time, despite commonly identifying emotional eating with overconsumption [3], emotional overeating is not the only option, and emotional undereating must be considered as well [4].

Nonetheless, emotional overeating is the most frequent situation, so emotional eating is commonly associated with increased consumption of energy-dense products [5,6], as well as excessive body mass and weight gain [7]. Moreover, it is indicated that emotional overeating may be motivated both by negative emotions (anxiety, sadness, loneliness, tiredness, anger) and by positive ones (happiness) [8]. At the same time, it should be emphasized that emotional eating may be BMI-dependent, and BMI-independent, while in extreme situations, emotional eating is associated with eating disorders and requires psychotherapeutic intervention [9]. Taking this into account, it has recently been recommended for obese individuals to be diagnosed in terms of emotional eating behavior, and if it is confirmed, to focus on the development of emotion regulation skills within the therapy [10].

It must be also emphasized that emotional eating may cause some adverse health effects as it has been positively associated with waist circumference [7] and body mass index (BMI) [11]. What is more, emotional eating may also be considered a risk factor for obesity, hypertension, or type 2 diabetes [12], so taking it into account, as well as the prevalence of emotional overeating as a coping mechanism in response to stress, should be an area of a particular interest.

During the Coronavirus Disease 2019 (COVID-19) pandemic, which was announced by the World Health Organization (WHO) [13], the nutritional behaviors of many populations changed. As indicated in a systematic review of longitudinal studies by González-Monroy et al. [14], the consumption of snacks, sweets, ultra-processed food products, and alcohol increased, while the consumption of fruits, vegetables, and fresh food products decreased. Some authors also suggest the role of negative emotions caused by stress and social isolation among various determinants of the observed situation [15].

Even though several studies assessed emotional overeating during the COVID-19 pandemic in the adult population, studies of children and adolescents are scarce. In the study by Philippe et al. [16], it was indicated that during the COVID-19 pandemic, boredom emotional overeating, and snacking between meals increased in children aged 3–12 years. Similarly, in the previous analysis conducted within the Polish Adolescents’ COVID-19 Experience (PLACE-19) Study [17], it was indicated that emotional eating was predicted by the female gender, and it contributed to excessive weight gain. Taking this into account, the present study aimed to assess emotional overeating background, including consumption in response to six emotions (anxiety, sadness, loneliness, tiredness, anger, and happiness), in the population of Polish adolescents within the PLACE-19 Study during the COVID-19 pandemic.

## 2. Materials and Methods

### 2.1. Ethical Background

The PLACE-19 Study was conducted in Polish secondary schools, based on the approval of the Ethics Committee of the Central Clinical Hospital of the Ministry of Interior and Administration in Warsaw (No. 2/2021) and in agreement with the Declaration of Helsinki. Both study participants and their parents/legal guardians provided informed consents for the study participation.

The PLACE-19 Study is a national Polish population-based study developed to describe the situation of Polish adolescents during the COVID-19 pandemic, including their hand hygiene and personal protective behaviors [18], nutritional behaviors [19,20], as well as food consumption psychological motivations [17,21]. The studied population was gathered upon recruitment based on a random quota sampling of secondary schools, so the population aged 15–20 years (the typical age of Polish secondary school students) was obtained. The Institute of Human Nutrition Sciences, Warsaw University of Life Sciences (WULS-SGGW) was in charge of the data gathering, management, and analysis within the PLACE-19 Study. 

### 2.2. PLACE-19 Study Population

The PLACE-19 Study was conducted within 3 phases, to assess hand hygiene and personal protective behaviors (first phase) [18], nutritional behaviors (second phase) [19,20], and food consumption psychological motivations (third phase) [17,21], while the presented analysis was conducted within the third phase, conducted from 21 January 2021 to 17 February 2021.

The studied population was gathered upon recruitment based on a random quota sampling of secondary schools, conducted based on administrative units of Poland—voivodeships and counties. From each of the 16 voivodeships, the random sample of 5 counties was chosen, and from each of the counties, the random sample of 5 secondary schools was chosen. The principals of randomly chosen secondary schools were invited to participate while receiving all the necessary details of the study. Finally, the principals of 19 schools agreed to participate in the study.

Afterwards, the principals invited their students to participate, while providing the students and their parents/legal guardians with all the necessary details. For those who agreed, the electronic link to a questionnaire was provided to complete the dedicated questionnaire, which did not allow gathering of any sensitive or personal data, but only those resulting from the aim of the study.

The inclusion criteria for the students were as follows:

- Student of the randomly sampled secondary school;
- Aged 15–20 years;
- Provided informed consent to participate;
- Provided informed consent of parents/legal guardians for participation (necessary only for students aged 15–18 years).

The exclusion criteria were as follows:

- Previous participation in the first or second phase of the PLACE-19 Study;
- Any missing data within the completed questionnaire;
- Any unreliable data within the completed questionnaire.

The final sample of 1126 students from randomly chosen secondary schools participated in the study, including 818 female and 308 male individuals, as presented previously [17]. The school response rate was 4.75%, and it was accompanied by a relatively high student response rate within schools of 40.9%. Such relatively low school response rate was typical for the studies conducted in Poland in the period of the COVID-19 pandemic [22]. It is explained by other authors as resulting from the fact that school principals and teachers did not want to have any interruption in their classes or their school to participate in such studies [23].

### 2.3. Questionnaire

Since the PLACE-19 Study was conducted during the COVID-19 pandemic, there were some periods when the secondary schools in Poland were in a model of remote education, and such a situation was observed during the third phase of the study. Based on the decision of the Polish Ministry of Education [24], all secondary schools in Poland had at-school education suspended. Taking this into account, the study was conducted using the method of the computer-assisted web interview (CAWI). According to Eurostat, the internet is widely available in Poland, as the share of households with internet access in 2021 was 92.42% [25]. As is indicated, internet access was slightly more prevalent in urban households, compared to rural households in 2021 (93.69% and 91.93%, respectively [25]), while in suburban households, the rate was 91.07% [25].

Emotional overeating was assessed while using the Emotional Overeating Questionnaire (EOQ), which was developed by Masheb and Grilo [8], and validated [26]. The Polish version of this questionnaire was developed for the study, as no such version was previously available. The questionnaire was translated and adapted for the Polish population based on the recommendations of the WHO [27]. The following stages were included:

- Forward translation to Polish (conducted by a Polish native-speaker researcher, fluent in English, and familiar with the studied area);
- Backward translation to English (conducted by a Polish native speaker, fluent in English, being the other independent researcher than for the previous stage, and not being familiar with the detailed aim of the study and questionnaire objectives);
- Expert panel (including Polish native speakers, fluent in English, being the other researchers than for the previous stages, to polish the final version of the questionnaire based on the results obtained within the previous stages, and to ensure semantic, idiomatic, cultural, and conceptual equivalence of the developed Polish version of the questionnaire).

The EOQ is the 6-item questionnaire, developed to be used as a self-reported form to assess overeating observed in response to specific emotions—anxiety, sadness, loneliness, tiredness, anger, and happiness, as presented by Masheb and Grilo [8]. For each emotion, the question asked is about how many days during the past 28 days (4 weeks) the respondent has eaten an unusually large amount of food, given the circumstances, in response to specific feelings. In order to make the questionnaire more understandable, each emotion is followed by its description (synonyms), as follows: anxiety: worry, jittery, nervous; sadness: blue, down, depressed; loneliness: bored, discouraged, worthless; tiredness: worn-out, fatigued; anger: upset, frustrated, furious; happiness: good, joyous, excited. In response, each respondent is asked to indicate the number of days while choosing an option from a 7-item scale, as follows: 0 days (rated as 0), 1–5 days (rated as 1), 6–12 days (rated as 2), 13–15 days (rated as 3), 16–22 days (rated as 4), 23–27 days (rated as 5), 28 days (rated as 6) [8].

While analyzing the responses provided by adolescents, the scores for each emotion were analyzed, while the highest score was attributed to a higher level of overeating (higher frequency of overeating). Additionally, the total score was calculated, by summing up the ratings for each emotion [8].

Within the questionnaire, additional questions were asked to verify the inclusion criteria, and to provide the general characteristics of the studied group, including questions about gender, body mass, body mass change during the COVID-19 pandemic, and declared tempting food products. 

The body mass was defined based on declared weight and height, which were used to calculate the body mass index (BMI), based on the standard calculation [28]. The results were interpreted as underweight, normal weight, overweight, or obesity, while a different approach was applied depending on age. For individuals aged at least 18 years (adults), the standard BMI cut-offs by WHO were applied, as follows: underweight attributed to a BMI < 18.5 kg/m^2^, normal weight attributed to a BMI of 18.5–24.9 kg/m^2^, overweight attributed to a BMI of 25.0–30.0 kg/m^2^, and obesity attributed to a BMI > 30.0 kg/m^2^ [28]. For individuals aged less than 18 years, the growth reference values were applied, while Polish gender-specific and age-specific values were chosen [29], and calculated using OLAF software (developed by Children’s Memorial Health Institute, Warsaw, Poland) [30], while the standard BMI growth reference value cut-offs by WHO were applied, as follows: underweight attributed to a BMI < 5th percentile, normal weight attributed to a BMI of 5th–85th percentile, overweight attributed to a BMI of 85th–95th percentile, obesity attributed to a BMI > 95th percentile [31]. 

Consequently, the body mass change during the COVID-19 pandemic was assessed based on the BMI change. The BMI before the pandemic was calculated based on the declared weight and height for March 2020, while the question was asked about weight and height before the remote education, as based on the decision of the Polish Ministry of Education [24], all secondary schools in Poland had at-school education suspended from this moment. Afterwards, the BMI values or BMI percentile values (depending on age) were compared to define losing weight, maintaining a stable weight, or gaining weight during the COVID-19 pandemic.

Additionally, declared tempting food products were assessed, based on the question adopted from the Self-Regulation of Eating Behaviour Questionnaire (SREBQ), by Kliemann et al. [32]. Within the multiple-choice question, the respondents were asked about the food products that they find tempting, while the following products were listed as suggested: chocolate, crisps, cakes, ice cream, bread/toast, fizzy drinks, biscuits, sweets, popcorn, pastries, pizza, fried foods, chips; other products were also allowed to be indicated, and no tempting food products were allowed to be declared. Based on the obtained individual lists for the respondents, they were classified as those indicating sweet products, salty products, both sweet and salty products, and indicating that they have no tempting products, as described previously [21].

### 2.4. Statistical Analysis

The following variables were studied as potentially associated with EOQ results: gender, body mass, body mass change during the COVID-19 pandemic, and tempting food products. The distribution of data was verified while using the Shapiro–Wilk test. The nonparametric tests were used, as follows: Mann–Whitney *U* test for comparison of 2 groups, Kruskal–Wallis analysis of variance (ANOVA) accompanied by post hoc Tukey test for comparison of more than 2 groups. Additionally, Spearman’s rank correlation coefficient and chi^2^ test were used. 

The statistical analysis was conducted using Statistica version 13.3 (StatSoft Inc., Tulsa, OK, USA), and *p* ≤ 0.05 was interpreted as a statistical significance. 

## 3. Results

The general characteristics of the group studied within the PLACE-19 Study, accompanied by the comparison between female and male respondents, is presented in Table 1. It was observed, that the body mass change during the COVID-19 pandemic differed between female and male participants, as a higher share of female participants declared losing weight, and a higher share of male participants declared no body mass change during the COVID-19 pandemic (*p* = 0.0015).

Emotional overeating assessed while using the EOQ in the group studied within the PLACE-19 Study, in sub-groups stratified by gender, is presented in Table 2. It was observed that emotional overeating during the COVID-19 pandemic differed between female and male participants, as female participants declared a higher frequency of overeating in response to feelings of anxiety (*p* = 0.0001), sadness (*p* < 0.0001), loneliness (*p* < 0.0001), and happiness (*p* = 0.0235), and were characterized by a higher total score (*p* < 0.0001).

Emotional overeating assessed while using the EOQ in the group studied within the PLACE-19 Study, in sub-groups stratified by body mass, is presented in Table 3. It was observed that emotional overeating during the COVID-19 pandemic differed between normal weight and obese participants, as obese participants declared a higher frequency of overeating in response to feelings of sadness (*p* = 0.0395), and loneliness (*p* = 0.0064).

Emotional overeating assessed while using the EOQ in the group studied within the PLACE-19 Study, in sub-groups stratified by body mass change during the COVID-19 pandemic, is presented in Table 4. It was observed that emotional overeating during the COVID-19 pandemic differed between participants losing weight and gaining weight, as participants gaining weight declared a higher frequency of overeating in response to feelings of anxiety (*p* < 0.0001), sadness (*p* < 0.0001), loneliness (*p* < 0.0001), tiredness (*p* = 0.0007), and anger (*p* < 0.0001), and were characterized by a higher total score (*p* < 0.0001) than participants losing weight or maintaining a stable weight. At the same time, participants gaining weight declared a higher frequency of overeating in response to feelings of happiness (*p* = 0.0189) than participants losing weight.

Emotional overeating assessed while using the EOQ in the group studied within the PLACE-19 Study, in sub-groups stratified by declared tempting food products, is presented in Table 5. It was observed that emotional overeating during the COVID-19 pandemic differed between participants declaring various tempting products, as participants declaring both sweet and salty products declared a higher frequency of overeating in response to feelings of anxiety (*p* < 0.0001), and sadness (*p* < 0.0001) than participants declaring no tempting products. Similarly, participants declaring both sweet and salty products declared a higher frequency of overeating in response to feelings of tiredness (*p* < 0.0001) than participants declaring only salty products, and those declaring no tempting products, and declared a higher frequency of overeating in response to feelings of happiness (*p* < 0.0001) than participants declaring only sweet products, and those declaring no tempting products. Participants declaring sweet products declared a higher frequency of overeating in response to feelings of anger (*p* < 0.0001) than participants declaring no tempting products. At the same time, participants declaring both sweet and salty products declared a higher frequency of overeating in response to feelings of loneliness (*p* < 0.0001), and were characterized by a higher total score (*p* < 0.0001) than all other respondents.

The presence of emotional overeating, during 28 days, assessed while using the EOQ in the group studied within the PLACE-19 Study, in sub-groups stratified by gender, is presented in Table 6. It was observed that emotional overeating during the COVID-19 pandemic differed between female and male participants, as female participants more often declared overeating in response to feelings of anxiety (*p* < 0.0001), sadness (*p* < 0.0001), loneliness (*p* < 0.0001), and happiness (*p* = 0.0002).

The presence of emotional overeating, during 28 days, assessed while using the EOQ in the group studied within the PLACE-19 Study, in sub-groups stratified by body mass, is presented in Table 7. It was observed that emotional overeating during the COVID-19 pandemic differed between obese and underweight participants, as obese participants more often declared overeating in response to feelings of loneliness (*p* = 0.0055).

The presence of emotional overeating, during 28 days, assessed while using the EOQ in the group studied within the PLACE-19 Study, in sub-groups stratified by body mass change during the COVID-19 pandemic, is presented in Table 8. It was observed that emotional overeating during the COVID-19 pandemic differed between participants gaining weight and those losing weight or maintaining stable body mass, as participants gaining weight more often declared overeating in response to feelings of anxiety (*p* < 0.0001), sadness (*p* < 0.0001), loneliness (*p* < 0.0001), tiredness (*p* = 0.0012), and anger (*p* < 0.0001).

The presence of emotional overeating, during 28 days, assessed while using the EOQ in the group studied within the PLACE-19 Study, in sub-groups stratified by declared tempting food products, is presented in Table 9. It was observed that emotional overeating during the COVID-19 pandemic differed between participants declaring both sweet and salty products and those declaring only sweet products, only salty products, or those declaring no tempting products, as participants declaring both sweet and salty products more often declared overeating in response to feelings of anxiety (*p* < 0.0001), sadness (*p* < 0.0001), loneliness (*p* < 0.0001), tiredness (*p* < 0.0001), anger (*p* < 0.0001), and happiness (*p* < 0.0001).

The correlation coefficients among emotional overeating variables assessed while using the EOQ in the group studied within the PLACE-19 Study is presented in Table 10. Within the studied group, the highest correlations were observed between total score and overeating in response to feelings of sadness (*p* < 0.0001; *R* = 0.74), tiredness (*p* < 0.0001; *R* = 0.75), and happiness (*p* < 0.0001; *R* = 0.74).

## 4. Discussion

The COVID-19 pandemic was a stressful period with unpleasant emotions intensified and pleasant emotions reduced [33]. The most prominent emotions for this period were worry and anger, but also sadness, and stress [34]. However, it was observed that emotional reactions did not reflect the pandemic course and did not increase with the virus spreading, but the life cycle of emotions was observed, as they increased, and independently from the pandemic situation, reverted to the previous level [35]. Independently from the natural life cycle of emotions, the experienced emotions influence mental health, and as it was observed during the COVID-19 pandemic in a systematic review by Burnatowska et al. [36] that many individuals were trying to cope with them by emotional eating.

As observed within the presented systematic review [36], such emotional eating was noted within the population of Polish adolescents during the COVID-19 pandemic, while the most influenced sub-groups and most affecting emotions may be defined. The previous observations indicating female adolescents, those of excessive body mass, and those gaining weight during the COVID-19 pandemic as those with the highest risk of emotional eating, measured by the Emotional Eater Questionnaire (EEQ) [17], are now confirmed within the presented study for the EOQ. 

Emotional eating, including especially negative overeating, was studied by some authors during the COVID-19 pandemic. Barcın-Güzeldere and Devrim-Lanpir [37] observed that COVID-related quarantine may be associated with emotional eating and resultant weight gain in an adult population, while participants with higher BMI presented more emotional eating behaviors. Madalı et al. [38] confirmed that during the COVID-19 pandemic, a higher frequency of emotional eating was observed in the sub-group of obese adult participants of the study than in normal weight and underweight ones. Yılmaz et al. [39] observed that during the COVID-19 pandemic, emotional eating increased in direct proportion with increasing BMI, and involuntary weight gain in an adult population. McAtamney et al. [40] indicated that adult respondents who were characterized by emotional eating behaviors increasing during the COVID-19 pandemic simultaneously demonstrated increasing depression levels. At the same time, Cecchetto et al. [41], in an adult population, indicated that during the COVID-19 pandemic, increased emotional eating was predicted by higher depression, and anxiety, but also by a higher quality of personal relationships, and quality of life.

At the same time, a novel observation was indicated in the present study, as the analysis of the association between declared tempting food products and emotional overeating allowed to indicate adolescents declaring both tempting sweet and salty products as those of the highest risk of emotional overeating, which was especially noticeable in comparison with those declaring no tempting products. It corresponds with the other observation that adolescents declaring both sweet and salty food products as tempting, during the COVID-19 pandemic, were characterized by a lower self-regulation of eating behaviors than others [21]. It may result from the general mechanism for food pleasure and food temptation, as tempting food products, independently from the fact that they are sweet or salty, cause within the brain the appearance of a reaction actively generated by a neuronal system, which is associated with pleasure and desire for the food [42]. A similar mechanism is involved in emotional eating, as it is associated with experiencing a positive feeling of gratification through the activation of brain reward pathways [43]. The role of declaring both tempting sweet and salty products may be explained as the body mass management strategies including temptation prevention strategies (to avoid exposure to tempting products), and resistance strategies (to overcome temptations, even if exposed to tempting products) [44], so in case of more tempting food products, the temptation prevention and resistance may be hindered.

While considering emotions most often causing emotional overeating, within the population of Polish adolescents during the COVID-19 pandemic, sadness and loneliness may be indicated as the most influential. Those emotions are negative ones (compared with happiness, being the only positive emotion within the EOQ [8]), which is in agreement with the narrow definition of emotional eating indicating the negative emotions as a stimulus [1]. However, emotional overeating in response to happiness, as a positive emotion, is also observed, but it is more common in respondents with binge-eating disorders than in those without [45], and it may explain why in the studied population group, this association was observed only in some analyses. 

The role of sadness and loneliness as emotions influencing emotional overeating in the studied population may have resulted from the period when the study was conducted, namely during the COVID-19 pandemic and resultant lockdowns, or social isolations. The loneliness of adolescents during the COVID-19 pandemic is the apparent consequence of the pandemic, being observed especially in adolescents experiencing depressive, aggression, or anxiety symptoms before the pandemic [46]. Moreover, the result of the other studies suggests that loneliness experienced during the COVID-19 pandemic may have influenced overconsumption [47]. Similarly, for sadness, the other studies indicated that patients with eating disorders declared increasing sadness levels during the COVID-19 pandemic [48], while sadness is indicated among the emotions significantly influencing emotional eating [49]. Taking this into account, the role of sadness and loneliness observed in the studied group of adolescents is not surprising.

In spite of the fact that novel interesting observations were noted within this study, its limitations should be listed as well. The most important limitation results from the applied tools, namely the questionnaires, in which each assessed variable is self-reported, and may result in the risk of bias. The other limitation is associated with a relatively low school response rate, even though it was accompanied by a relatively high student response rate within schools, which also may have influenced the obtained results. Last but not least, it should be noted that the study was conducted only in Poland, so further analysis for other countries should be similarly conducted.

## 5. Conclusions

The sub-groups with the highest frequency of emotional overeating were the female respondents, obese participants, those gaining weight, and those declaring both sweet and salty products as tempting, while among the emotions most often causing emotional overeating, there were sadness and loneliness.

## Figures and Tables

**Table 1:** The general characteristics of the group studied within the Polish Adolescents’ COVID-19 Experience (PLACE-19) Study, accompanied by the comparison between female and male respondents.

		Total *n* = 1126	Female *n* = 818	Male *n* = 308	*p*-Value
Age (years)	Mean ± SD	16.7 ± 1.1	16.7 ± 1.1	16.7 ± 1.1	0.4109 ^2^
	Median (25th–75th)	17.0 ^1^ (16.0–17.0)	17.0 ^1^ (16.0–18.0)	16.5 ^1^ (16.0–17.0)	
Body mass	Underweight	39 (3.5%)	29 (3.5%)	10 (3.2%)	0.4120 ^3^
	Normal weight	842 (74.8%)	619 (75.7%)	223 (72.4%)	
	Overweight	158 (14.0%)	106 (13.0%)	52 (16.9%)	
	Obesity	87 (7.7%)	64 (7.8%)	23 (7.5%)	
Body mass change	Lost weight	296 (26.3%)	238 (29.1%)	58 (18.8%)	0.0015 ^3^
	No body mass change	519 (46.1%)	357 (43.6%)	162 (52.6%)	
	Gained weight	311 (27.6%)	223 (27.3%)	88 (28.6%)	
Tempting food products	Sweet products	105 (9.3%)	77 (9.4%)	28 (9.1%)	0.6896 ^3^
	Salty products	71 (6.3%)	54 (6.6%)	17 (5.5%)	
	Sweet and salty products	863 (76.6%)	628 (76.8%)	235 (76.3%)	
	No tempting products declared	87 (7.7%)	59 (7.2%)	28 (9.1%)	

^1^ nonparametric distribution (verified using Shapiro–Wilk test; *p* ≤ 0.05); ^2^ compared using Mann–Whitney *U* test; ^3^ compared using chi^2^ test.

**Table 2:** Emotional overeating assessed while using the Emotional Overeating Questionnaire (EOQ) in the group studied within the Polish Adolescents’ COVID-19 Experience (PLACE-19) Study, stratified by gender.

	Total *n* = 1126		Female *n* = 818		Male *n* = 308		*p*-Value ^2^
	Mean (SD)	Median (IQR)	Mean (SD)	Median (IQR)	Mean (SD)	Median (IQR)	
Anxiety	0.57 (1.03)	0.0 ^1^ (1.0)	0.61 (1.02)	0.0 ^1^ (1.0)	0.47 (1.06)	0.0 ^1^ (1.0)	0.0001
Sadness	0.77 (1.18)	0.0 ^1^ (1.0)	0.85 (1.17)	1.0 ^1^ (1.0)	0.55 (1.18)	0.0 ^1^ (1.0)	<0.0001
Loneliness	0.87 (1.35)	0.0 ^1^ (1.0)	0.96 (1.37)	0.0 ^1^ (1.0)	0.66 (1.28)	0.0 ^1^ (1.0)	<0.0001
Tiredness	0.80 (1.21)	0.0 ^1^ (1.0)	0.78 (1.17)	0.0 ^1^ (1.0)	0.87 (1.32)	0.0 ^1^ (1.0)	0.6417
Anger	0.56 (1.06)	0.0 ^1^ (1.0)	0.58 (1.04)	0.0 ^1^ (1.0)	0.53 (1.11)	0.0 ^1^ (1.0)	0.0892
Happiness	1.25 (1.55)	1.0 ^1^ (2.0)	1.25 (1.47)	1.0 ^1^ (1.0)	1.23 (1.76)	0.0 ^1^ (2.0)	0.0235
Total score	0.80 (0.92)	0.5 ^1^ (1.2)	0.84 (0.88)	0.7 ^1^ (1.0)	0.72 (1.04)	0.3 ^1^ (1.0)	<0.0001

^1^ nonparametric distribution (verified using Shapiro–Wilk test; *p* ≤ 0.05); ^2^ compared using Mann–Whitney *U* test.

**Table 3:** Emotional overeating assessed while using the Emotional Overeating Questionnaire (EOQ) in the group studied within the Polish Adolescents’ COVID-19 Experience (PLACE-19) Study, stratified by body mass.

	Underweight *n* = 39		Normal Weight *n* = 842		Overweight *n* = 158		Obesity *n* = 87		*p*-Value ^2^
	Mean (SD)	Median (IQR)	Mean (SD)	Median (IQR)	Mean (SD)	Median (IQR)	Mean (SD)	Median (IQR)	
Anxiety	0.51 (1.19)	0.0 ^1^ (1.0)	0.53 (0.96)	0.0 ^1^ (1.0)	0.68 (1.22)	0.0 ^1^ (1.0)	0.77 (1.29)	0.0 ^1^ (1.0)	0.2322
Sadness	0.79 (1.43)	0.0 ^1^ (1.0) ^ab^	0.71 (1.09)	0.0 ^1^ (1.0) ^a^	0.93 (1.41)	0.0 ^1^ (1.0) ^ab^	1.05 (1.36)	1.0 ^1^ (1.0) ^b^	0.0395
Loneliness	0.77 (1.48)	0.0 ^1^ (1.0) ^ab^	0.82 (1.30)	0.0 ^1^ (1.0) ^a^	0.99 (1.47)	0.0 ^1^ (2.0) ^ab^	1.23 (1.50)	1.0 ^1^ (2.0) ^b^	0.0064
Tiredness	0.77 (1.16)	0.0 ^1^ (1.0)	0.78 (1.18)	0.0 ^1^ (1.0)	0.87 (1.32)	0.0 ^1^ (1.0)	0.93 (1.39)	0.0 ^1^ (1.0)	0.8302
Anger	0.51 (1.17)	0.0 ^1^ (1.0)	0.55 (1.01)	0.0 ^1^ (1.0)	0.61 (1.20)	0.0 ^1^ (1.0)	0.60 (1.21)	0.0 ^1^ (1.0)	0.8835
Happiness	1.44 (1.82)	1.0 ^1^ (3.0)	1.24 (1.55)	1.0 ^1^ (2.0)	1.28 (1.58)	1.0 ^1^ (2.0)	1.16 (1.39)	1.0 ^1^ (2.0)	0.9915
Total score	0.80 (1.12)	0.5 ^1^ (1.2)	0.77 (0.87)	0.5 ^1^ (1.2)	0.89 (1.13)	0.7 ^1^ (1.2)	0.96 (1.13)	0.7 ^1^ (1.2)	0.4144

^1^ nonparametric distribution (verified using Shapiro–Wilk test; *p* ≤ 0.05); ^2^ compared using Kruskal–Wallis analysis of variance (ANOVA) accompanied by post hoc Tukey test, values with different letters (a, b) differ in rows.

**Table 4:** Emotional overeating assessed while using the Emotional Overeating Questionnaire (EOQ) in the group studied within the Polish Adolescents’ COVID-19 Experience (PLACE-19) Study, stratified by body mass change during the COVID-19 pandemic.

	Lost Weight *n* = 296		No Body Mass Change *n* = 519		Gained Weight *n* = 311		*p*-Value ^2^
	Mean (SD)	Median (IQR)	Mean (SD)	Median (IQR)	Mean (SD)	Median (IQR)	
Anxiety	0.46 (0.94)	0.0 ^1^ (1.0) ^a^	0.48 (0.96)	0.0 ^1^ (1.0) ^a^	0.82 (1.19)	0.0 ^1^ (1.0) ^b^	<0.0001
Sadness	0.70 (1.13)	0.0 ^1^ (1.0) ^a^	0.64 (1.11)	0.0 ^1^ (1.0) ^a^	1.05 (1.29)	1.0 ^1^ (1.0) ^b^	<0.0001
Loneliness	0.76 (1.20)	0.0 ^1^ (1.0) ^a^	0.72 (1.26)	0.0 ^1^ (2.0) ^a^	1.23 (1.54)	1.0 ^1^ (1.0) ^b^	<0.0001
Tiredness	0.72 (1.19)	0.0 ^1^ (1.0) ^a^	0.72 (1.10)	0.0 ^1^ (1.0) ^a^	1.03 (1.38)	1.0 ^1^ (1.0) ^b^	0.0007
Anger	0.54 (1.10)	0.0 ^1^ (1.0) ^a^	0.47 (0.99)	0.0 ^1^ (1.0) ^a^	0.75 (1.15)	0.0 ^1^ (1.0) ^b^	<0.0001
Happiness	1.07 (1.44)	1.0 ^1^ (2.0) ^a^	1.26 (1.61)	1.0 ^1^ (2.0) ^ab^	1.38 (1.55)	1.0 ^1^ (2.0) ^b^	0.0189
Total score	0.71 (0.91)	0.4 ^1^ (1.0) ^a^	0.71 (0.91)	0.5 ^1^ (1.2) ^a^	1.04 (0.91)	0.8 ^1^ (1.0) ^b^	<0.0001

^1^ nonparametric distribution (verified using Shapiro–Wilk test; *p* ≤ 0.05); ^2^ compared using Kruskal–Wallis analysis of variance (ANOVA) accompanied by post hoc Tukey test, values with different letters (a, b) differ in rows.

**Table 5:** Emotional overeating assessed while using the Emotional Overeating Questionnaire (EOQ) in the group studied within the Polish Adolescents’ COVID-19 Experience (PLACE-19) Study, stratified by declared tempting food products.

	Sweet Products *n* = 105		Salty Products *n* = 71		Sweet and Salty Products *n* = 863		No Tempting Products Declared *n* = 87		*p*-Value ^2^
	Mean (SD)	Median (IQR)	Mean (SD)	Median (IQR)	Mean (SD)	Median (IQR)	Mean (SD)	Median (IQR)	
Anxiety	0.37 (0.75)	0.0 ^1^ (0.0) ^ab^	0.46 (1.09)	0.0 ^1^ (1.0) ^ab^	0.64 (1.08)	0.0 ^1^ (1.0) ^a^	0.21 (0.73)	0.0 ^1^ (0.0) ^b^	<0.0001
Sadness	0.70 (1.11)	0.0 ^1^ (1.0) ^ab^	0.51 (1.12)	0.0 ^1^ (1.0) ^ac^	0.84 (1.20)	0.0 ^1^ (1.0) ^b^	0.29 (0.85)	0.0 ^1^ (0.0) ^c^	<0.0001
Loneliness	0.65 (1.18)	0.0 ^1^ (1.0) ^a^	0.48 (0.95)	0.0 ^1^ (1.0) ^a^	0.99 (1.41)	0.0 ^1^ (1.0) ^b^	0.31 (0.85)	0.0 ^1^ (0.0) ^a^	<0.0001
Tiredness	0.55 (0.84)	0.0 ^1^ (1.0) ^ab^	0.54 (1.07)	0.0 ^1^ (1.0) ^a^	0.90 (1.27)	0.0 ^1^ (1.0) ^b^	0.38 (0.93)	0.0 ^1^ (0.0) ^a^	<0.0001
Anger	0.38 (0.87)	0.0 ^1^ (0.0) ^a^	0.45 (0.98)	0.0 ^1^ (1.0) ^ab^	0.63 (1.10)	0.0 ^1^ (1.0) ^ab^	0.24 (0.82)	0.0 ^1^ (0.0) ^b^	<0.0001
Happiness	0.79 (1.14)	0.0 ^1^ (1.0) ^a^	1.07 (1.62)	0.0 ^1^ (1.0) ^ab^	1.37 (1.60)	1.0 ^1^ (2.0) ^b^	0.66 (1.20)	0.0 ^1^ (1.0) ^a^	<0.0001
Total score	0.57 (0.73)	0.3 ^1^ (0.8) ^a^	0.58 (0.89)	0.2 ^1^ (1.0) ^a^	0.9 (0.96)	0.7 ^1^ (1.2) ^b^	0.35 (0.67)	0.0 ^1^ (0.5) ^a^	<0.0001

^1^ nonparametric distribution (verified using Shapiro–Wilk test; *p* ≤ 0.05); ^2^ compared using Kruskal–Wallis analysis of variance (ANOVA) accompanied by post hoc Tukey test, values with different letters (a, b, c) differ in rows.

**Table 6:** The presence of emotional overeating, during 28 days, assessed while using the Emotional Overeating Questionnaire (EOQ) in the group studied within the Polish Adolescents’ COVID-19 Experience (PLACE-19) Study, stratified by gender.

During 28 Days		Total *n* = 1126	Female *n* = 818	Male *n* = 308	*p*-Value ^1^
Anxiety	Not eating in response to emotion	718 (63.8%)	493 (60.3%)	225 (73.1%)	<0.0001
	Eating in response to emotion at least once	408 (36.2%)	325 (39.7%)	83 (26.9%)	
Sadness	Not eating in response to emotion	625 (55.5%)	407 (49.8%)	218 (70.8%)	<0.0001
	Eating in response to emotion at least once	501 (44.5%)	411 (50.2%)	90 (29.2%)	
Loneliness	Not eating in response to emotion	635 (56.4%)	424 (51.8%)	211 (68.5%)	<0.0001
	Eating in response to emotion at least once	491 (43.6%)	394 (48.2%)	97 (31.5%)	
Tiredness	Not eating in response to emotion	619 (55.0%)	450 (55.0%)	169 (54.9%)	0.9695
	Eating in response to emotion at least once	507 (45.0%)	368 (45.0%)	139 (45.1%)	
Anger	Not eating in response to emotion	747 (66.3%)	530 (64.8%)	217 (70.5%)	0.0731
	Eating in response to emotion at least once	379 (33.7%)	288 (35.2%)	91 (29.5%)	
Happiness	Not eating in response to emotion	481 (42.7%)	322 (39.4%)	159 (51.6%)	0.0002
	Eating in response to emotion at least once	645 (57.3%)	496 (60.6%)	149 (48.4%)	

^1^ compared using chi^2^ test.

**Table 7:** The presence of emotional overeating, during 28 days, assessed while using the Emotional Overeating Questionnaire (EOQ) in the group studied within the Polish Adolescents’ COVID-19 Experience (PLACE-19) Study, stratified by body mass.

During 28 Days		Underweight *n* = 39	Normal Weight *n* = 842	Overweight *n* = 158	Obesity *n* = 87	*p*-Value ^1^
Anxiety	Not eating in response to emotion	29 (74.4%)	542 (64.4%)	97 (61.4%)	50 (57.5%)	0.2720
	Eating in response to emotion at least once	10 (25.6%)	300 (35.6%)	61 (38.6%)	37 (42.5%)	
Sadness	Not eating in response to emotion	23 (59.0%)	481 (57.1%)	84 (53.2%)	37 (42.5%)	0.0611
	Eating in response to emotion at least once	16 (41.0%)	361 (42.9%)	74 (46.8%)	50 (57.5%)	
Loneliness	Not eating in response to emotion	25 (64.1%)	489 (58.1%)	87 (55.1%)	34 (39.1%)	0.0055
	Eating in response to emotion at least once	14 (35.9%)	353 (41.9%)	71 (44.9%)	53 (60.9%)	
Tiredness	Not eating in response to emotion	20 (51.3%)	469 (55.7%)	85 (53.8%)	45 (51.7%)	0.8365
	Eating in response to emotion at least once	19 (48.7%)	373 (44.3%)	73 (46.2%)	42 (48.3%)	
Anger	Not eating in response to emotion	28 (71.8%)	553 (65.7%)	108 (68.4%)	58 (66.7%)	0.8069
	Eating in response to emotion at least once	11 (28.2%)	289 (34.3%)	50 (31.6%)	29 (33.3%)	
Happiness	Not eating in response to emotion	18 (46.2%)	361 (42.9%)	70 (44.3%)	32 (36.8%)	0.6567
	Eating in response to emotion at least once	21 (53.8%)	481 (57.1%)	88 (55.7%)	55 (63.2%)	

^1^ compared using chi^2^ test.

**Table 8:** The presence of emotional overeating, during 28 days, assessed while using the Emotional Overeating Questionnaire (EOQ) in the group studied within the Polish Adolescents’ COVID-19 Experience (PLACE-19) Study, stratified by body mass change during the COVID-19 pandemic.

During 28 Days		Lost Weight *n* = 296	No Body Mass Change *n* = 519	Gained Weight *n* = 311	*p*-Value ^1^
Anxiety	Not eating in response to emotion	205 (69.3%)	354 (68.2%)	159 (51.1%)	<0.0001
	Eating in response to emotion at least once	91 (30.7%)	165 (31.8%)	152 (48.9%)	
Sadness	Not eating in response to emotion	176 (59.5%)	320 (61.7%)	129 (41.5%)	<0.0001
	Eating in response to emotion at least once	120 (40.5%)	199 (38.3%)	182 (58.5%)	
Loneliness	Not eating in response to emotion	174 (58.8%)	324 (62.4%)	137 (44.1%)	<0.0001
	Eating in response to emotion at least once	122 (41.2%)	195 (37.6%)	174 (55.9%)	
Tiredness	Not eating in response to emotion	180 (60.8%)	294 (56.6%)	145 (46.6%)	0.0012
	Eating in response to emotion at least once	116 (39.2%)	225 (43.4%)	166 (53.4%)	
Anger	Not eating in response to emotion	206 (69.6%)	371 (71.5%)	170 (54.7%)	<0.0001
	Eating in response to emotion at least once	90 (30.4%)	148 (28.5%)	141 (45.3%)	
Happiness	Not eating in response to emotion	141 (47.6%)	221 (42.6%)	119 (38.3%)	0.0655
	Eating in response to emotion at least once	155 (52.4%)	298 (57.4%)	192 (61.7%)	

^1^ compared using chi^2^ test.

**Table 9:** The presence of emotional overeating, during 28 days, assessed while using the Emotional Overeating Questionnaire (EOQ) in the group studied within the Polish Adolescents’ COVID-19 Experience (PLACE-19) Study, stratified by declared tempting food products.

During 28 Days		Sweet Products *n* = 105	Salty Products *n* = 71	Sweet and Salty Products *n* = 863	No Tempting Products Declared *n* = 87	*p*-Value ^1^
Anxiety	Not eating in response to emotion	79 (75.2%)	50 (70.4%)	513 (59.4%)	76 (87.4%)	<0.0001
	Eating in response to emotion at least once	26 (24.8%)	21 (29.6%)	350 (40.6%)	11 (12.6%)	
Sadness	Not eating in response to emotion	61 (58.1%)	49 (69.0%)	444 (51.4%)	71 (81.6%)	<0.0001
	Eating in response to emotion at least once	44 (41.9%)	22 (31.0%)	419 (48.6%)	16 (18.4%)	
Loneliness	Not eating in response to emotion	72 (68.6%)	49 (69.0%)	445 (51.6%)	69 (79.3%)	<0.0001
	Eating in response to emotion at least once	33 (31.4%)	22 (31.0%)	418 (48.4%)	18 (20.7%)	
Tiredness	Not eating in response to emotion	68 (64.8%)	49 (69.0%)	434 (50.3%)	68 (78.2%)	<0.0001
	Eating in response to emotion at least once	37 (35.2%)	22 (31.0%)	429 (49.7%)	19 (21.8%)	
Anger	Not eating in response to emotion	83 (79.0%)	50 (70.4%)	540 (62.6%)	74 (85.1%)	<0.0001
	Eating in response to emotion at least once	22 (21.0%)	21 (29.6%)	323 (37.4%)	13 (14.9%)	
Happiness	Not eating in response to emotion	59 (56.2%)	37 (52.1%)	329 (38.1%)	56 (64.4%)	<0.0001
	Eating in response to emotion at least once	46 (43.8%)	34 (47.9%)	534 (61.9%)	31 (35.6%)	

^1^ compared using chi^2^ test.

**Table 10:** The correlation coefficients among emotional overeating variables assessed while using the Emotional Overeating Questionnaire (EOQ) in the group studied within the Polish Adolescents’ COVID-19 Experience (PLACE-19) Study.

Variables	Anxiety ^1^	Sadness ^1^	Loneliness ^1^	Tiredness ^1^	Anger ^1^	Happiness ^1^	Total Score ^1^
Anxiety	1						
Sadness	0.63	1					
Loneliness	0.50	0.65	1				
Tiredness	0.52	0.45	0.44	1			
Anger	0.53	0.52	0.42	0.51	1		
Happiness	0.37	0.36	0.35	0.52	0.41	1	
Total score	0.70	0.74	0.72	0.75	0.67	0.74	1

^1^ *p* < 0.0001 for Spearman’s rank correlation coefficient.

## Data Availability

The data presented in this study are available on request from the corresponding author.
